# Procedure for the Screening of Eggs and Egg Products to Detect Oxolonic Acid, Ciprofloxacin, Enrofloxacin, and Sarafloxacin Using Micellar Liquid Chromatography

**DOI:** 10.3390/antibiotics8040226

**Published:** 2019-11-15

**Authors:** Juan Peris-Vicente, Daniel García-Ferrer, Pooja Mishra, Jaume Albiol-Chiva, Abhilasha Durgbanshi, Samuel Carda-Broch, Devasish Bose, Josep Esteve-Romero

**Affiliations:** 1 Department of Analytical Chemistry, Faculty of Chemistry, Universitat de València, 46100 Burjassot, Spain; juan.peris@uv.es (J.P.-V.); danielgarciaferrer@hotmail.com (D.G.-F.); 2 Laboratory of Clinical Analysis, General University Hospital of de Castelló, 12004 Castelló, Spain; 3 Department of Chemistry, Doctor Harisingh Gour Vishwavidyalaya (A Central University), Madhya Pradesh 470003, India; mishrapooja2013@gmail.com (P.M.); abhiasha126@gmail.com (A.D.); 4 Bioanalytical Chemistrya, Department of Physical and Analytical Chemistrya, ESTCE, Universitat Jaume I, 12071 Castelló, Spain; albiolj@uji.es (J.A.-C.); scarda@uji.es (S.C.-B.); 5 Department of Criminology and Forensic Science, Doctor Harisingh Gour Vishwavidyalaya (A Central University), Madhya Pradesh 470003, India; devonebose@gmail.com

**Keywords:** food safety, fluorescence, laying hen, optimization, solid-to-liquid extraction, validation

## Abstract

A method based on micellar liquid chromatography was developed to determine oxolinic acid, ciprofloxacin, enrofloxacin, and sarafloxacin in eggs and egg products. The antimicrobial drugs were obtained in a micellar solution which was directly injected. The analytes were resolved using a C18 column and a mobile phase of 0.05 M sodium dodecyl sulfate—7.5% 1-propanol—0.5% triethylamine, buffered at pH 3 with phosphate salt, running under the isocratic mode. The signal was monitored by fluorescence. Validation was successfully performed according to the EU Commission Decision 2002/657/EC in terms of specificity, calibration range (LOQ to 1 mg/kg), linearity (*R*^2^ > 0.9991), limit of detection and decision limit (0.01–0.05 mg/kg), limit of quantification (0.025–0.150 mg/kg), detection capability (<0.4 times decision limit), trueness (−14.2% to +9.8%), precision (<14.0%), robustness, and stability. The procedure was environmentally friendly, safe, easy-to-conduct, inexpensive, and had a high sample throughput, thus it is useful for routine analysis as a screening method in a laboratory for food residue control.

## 1. Introduction

Antibiotics refer to a large category of drugs used to kill or avoid the growth of bacterial microorganisms. In human medicine, they are prescribed to cure microbial infections. In husbandry, they are administered for the same reasons but also unethically for prophylaxis purposes and to promote growth, increasing the economic profit of a farm [1,2]. Quinolones are among the most important antibacterial agents used in human medicine and are active against both Gram-positive and Gram-negative bacteria through the inhibition of their DNA gyrase. They are well absorbed after oral administration and extensively distributed in tissues, and they display stable and favorable pharmacokinetics and dose-dependent activity. Such characteristics make these drugs suitable to act as therapy for a large number of infections on animal farms [3]. However, inappropriate or abusive use of antibiotics in poultry farms (or by poultry producers) might provoke the transfer and accumulation of their residues in food products. This causes exposure to the consumer to these compounds in low concentrations which may produce toxic reactions and stimulate the emergence of quinolone-resistant pathogens [4,5]. It has been largely proven that eggs from poultry treated with pharmaceutical products contain drug residues, even those laid days to weeks after treatment cessation [6].

Nowadays, there is a worldwide concern among society and public agencies about the dramatic consequences of quinolone misuse in population health [7]. In the frame of food safety, the European Union (EU) has regulated the use of quinolones as veterinary drugs in food-producing animals and has established maximum residue limits (MRLs) of antibiotics in food. According to EU regulations, quinolones, such as ciprofloxacin (CIPRO), enrofloxacin (ENRO), sarafloxacin (SARA), and oxolinic acid (OXO), are “not for use in animals from which eggs are produced for human consumption”, which means they are strictly forbidden in rearing laying hens. No MRLs have been stated for them, and eggs and industrially elaborated egg products containing quinolone residues at any level must be rejected [8,9]. Accordingly, their residues need to be controlled to verify the compliance of producers and importers with the regulation. 

The determination of quinolones in complex food samples requires a previous extraction step of the analyte from the matrix, followed by different clean-up steps that involve liquid–liquid extraction (LLE) [10,11], solid-phase extraction (SPE) [12,13], dialysis [14] or supercritical fluid extraction (SFE) [15]. A comprehensive review of the current trends in sample preparation to isolate veterinary drugs and growth promoters from foods is presented in Reference [16]. Turbulent-flow chromatography coupled with tandem mass spectrometry (TFC-LC-MS/MS) is a technique that eliminates time-consuming simple clean-up steps, increases productivity, and reduces solvent consumption without harming sensitivity or productivity.

The most recent studies on antibiotics in eggs have been published using high-performance liquid chromatography/tandem mass spectrometry (HPLC-MS/MS) [17,18,19,20,21,22,23,24]. However, this equipment is very expensive and only a few laboratories can afford it. Other methods involve HPLC with fluorescence (FLD) detection [25,26,27]. As previously commented, one of the main problems in multiresidue antibiotic analyses in incurred samples is the extraction; cleanup and preconcentration of the matrix analytes before the instrumental analysis are excessively tedious and complex.

Micellar liquid chromatography (MLC) [28] is an attractive alternative to conventional HPLC methods that use a surfactant solution above the critical micellar concentration instead of aqueous-organic solvents as mobile phases. The most common surfactant is the anionic sodium dodecyl sulfate (SDS). It has previously been used for the determination of quinolones in a large variety of food matrices with direct injection from liquid samples and by simple solid-to-liquid extraction followed by direct injection of the supernatant from solid samples. Micellar liquid chromatography allows the analysis of physico-chemically complex food matrices, usually without the aid of a purification step thus considerably reducing the cost, manipulation, use of hazardous and volatile organic solvents, and analysis time. Indeed, SDS-micelles and SDS-monomers tend to bind proteins and other biomacromolecules competitively, provoking its denaturing (in the case of proteins) and solubilization. Therefore, they are washed harmlessly away to elute with the solvent front rather than precipitating into the column, reducing the probabilities of overlapping with the analytes. Besides, protein-bound drugs are released and can be determined [7,29,30,31,32].

The aim of this work was to develop a multiresidue HPLC procedure with micellar mobile phases to simultaneously determine several quinolones (ciprofloxacin, enrofloxacin, sarafloxacin, and oxolinic acid) in fresh eggs and industrially made egg products: boiled egg; liquid pasteurized egg white and yolk; powdered whole egg; powdered egg white and yolk; and omelets. The method was validated in line with the Commission Decision 2002/657/EC [33] in terms of selectivity, linearity, decision limit, detection capability, precision, and robustness. The proposed method was applied to determine these compounds in a large number of incurred samples of egg and egg product samples, distributed in retail shops to consumers.

## 2. Experimental

### 2.1. Standards and Reagents

Powdered standards of oxolinic acid (purity > 97.0%), enrofloxacin (>98.0%), ciprofloxacin (>98%), and sarafloxacin (>97.2%) were bought from Sigma–Aldrich (St-Louis, MO, USA). Sodium dodecyl sulphate (>98.0%) and sodium dihydrogen phosphate monohydrate (>99.0%) were from Scharlab (Barcelona, Spain). The HPLC grade 1-propanol was purchased from Merck KGaA (Darmstadt, Germany). Hydrochloric acid (37.0%), ethanol (HPLC grade), and triethylamine (>99.5%) were supplied by J.T. Baker (Deventer, The Netherlands). Ultrapure water was in-lab produced from deionized water (provided by the University as tap water) using an ultrapure generator device Simplicity UV (Millipore S.A.S., Molsheim, France). All the aqueous solutions were prepared using freshly made ultrapure water. The ultrapure water was not stored.

### 2.2. Preparation of Solutions

The micellar solutions were made as follows: the proper quantity of SDS and NaH_2_PO_4_∙H_2_O were weighed and solved in ultrapure water using a magnetic stirrer. Later, the adequate volume of triethylamine (TEA) was added, and the pH was adjusted by adding drops of an HCl solution. Afterwards, the appropriate volume of 1-propanol was introduced to obtain the desired proportion, and then the flask was filled with ultrapure water. Finally, the solution was ultrasonicated to ensure solubilization and filtered with the aid of a vacuum pump through a 0.45 µm membrane filter (Micron Separations, Westboro, MA, USA) placed on a Büchner funnel.

Stock solutions of the quinolones (100 mg/L) were made by solving the appropriate amount of the powdered standard in 5% of ethanol in a volumetric flask, which was then filled up with a solution of 0.05 M SDS buffered at pH 3. These solutions were ultrasonicated to achieve solubilization. Working solutions were made by consecutive dilution of the stock solutions in the same micellar solution. All the standard and stock solutions were stored at +4 °C in amber vials for a maximum of two months. Before their utilization, these stored solutions were warmed at room temperature until the completion of the dissolution of the crystals of SDS that were formed overnight.

### 2.3. Chromatographic Conditions

An HP1100 (Agilent Technologies, Palo Alto, CA, USA) equipped with an isocratic pump, a degasser, an autosampler, a 20 μL loop, and a fluorescence detector was used for the chromatographic analyses. No module worked at a regulated temperature. The software Chemstation Rev.A.10.01 (Agilent Technologies, California, CA, USA) was used to control the instrumentation as well as to register and process the signal. The dead time (t_0_ ≈ 1.0 min) and the retention time (t_R_) were directly taken from the chromatogram. We calculated the retention factor (*k*) [34], efficiency (N, number of theoretical plates), asymmetry (B/A), and resolution (Rs) [35].

The column was a C18 Kromasil (Scharlab, Barcelona, Spain) with the following features: length, 150 mm; internal diameter, 4.6 mm; particle size, 5 µm; pore size, 10 nm. The mobile phase was an aqueous solution of 0.05 M SDS—7.5% 1-propanol—0.5% triethylamine, buffered at pH 3 with 0.01 M phosphate salt, running at 1 mL/min under isocratic mode. The detection was performed by fluorescence, applying the following excitation/emission wavelength program: 0–10, 260/366 nm, and 10.01 to 30: 280–455 nm. The working and cleaning specifications regarding the utilization of the chromatographic instrumentation when using micellar mobile phases are described in Reference [36].

All the samples to be injected were filtered through a 0.45-μm Nylon membrane filter, with the aid of a 3 mL syringe, before introduction into the vials.

### 2.4. Sample Processing

Fresh eggs and industrially made egg products (i.e., boiled egg, pasteurized egg white and yolk, powdered whole egg, powdered egg white and yolk, and omelet) were purchased in local supermarkets. Whites and yolks were separated for fresh and boiled eggs. 

The liquid samples (fresh and pasteurized egg yolks and whites) were kept in the freezer at −20 °C. The solid samples (boiled egg yolk and whites; powdered whole egg; powdered egg whites and yolk; and omelet) were finely ground using a mincer (Model MZ10, Petra Electric, Burgau, Germany) at 5000 rpm for 5 min and kept in the freezer at −20 °C. All the samples were stored a maximum of 2 months and thawed the day of the analysis until reaching room temperature.

Liquid samples were homogenized for 10 min through shaking using a magnetic stirrer. An aliquot was 1/5 *w*/*v*, diluted in mobile phase, filtered, and directly injected. For spiked samples, the appropriate volume of quinolone standard was added before the dilution. 

A stirring batch solid-to-liquid extraction [37] (SBSLE) procedure was used to extract the quinolones from the solid samples [7,32]. Five grams were introduced in an Erlenmeyer flask containing 50 mL of mobile phase; the solution was shaken in a magnetic stirrer for 1 h and ultrasonicated for 15 min. The supernatant was decanted, filtered through a 0.45-μm Nylon membrane filter using a Büchner funnel with the help of a vacuum pump and injected. For fortified samples, the proper amount of quinolone standard solution was injected into the crushed sample, and the mixture was left for 24 h at room temperature to stimulate a slow elimination of the solvent and maximize the integration of the matrix and the analytes [32]. Therefore, these artificially contaminated samples were similar to the incurred ones. 

## 3. Results and Discussion

### 3.1. Optimization of the Chromatographic Conditions

General chromatographic conditions were taken from previously published methods about the determination of quinolone residues in food matrices such as fish flesh [29], honey [30,31], and meat [7,32]: flow rate, 1 mL/min; isocratic mode; non-controlled temperature; stationary phase, C18; surfactant, SDS; buffer, 0.01 M phosphate salt; pH, 3; sacrificial base, TEA at 0.5%; and the optimal values of fluorescence excitation/emission wavelength. The wavelength program used to register the signal was designed to determine each quinolone at their optimal detection conditions. Under these conditions, OXO is neutral and ENRO, CIPRO, and SARA, positively monocharged. Oxolinic acid exhibits low retention and requires a hybrid mobile phases with limited elution strength. Therefore, 1-propanol was selected as the organic solvent. In the context of this work, we optimized the concentration of SDS and 1-propanol in the hybrid mobile phase to determine OXO, ENRO, CIPRO, and SARA with a maximum of resolution, peak shape and the minimum analysis time.

We have previously demonstrated that the retention factor and efficiency of the quinolones using hybrid mobile phases decreases at increasing concentrations of SDS, as the quinolones bind to the micelles [7,32]. Indeed, the number of micelles is directly related to the total concentration of SDS. In order to maximize the peak shape, the concentration of SDS was fixed to the minimal value recommended in MLC, 0.05 M.

A standard solution of 0.1 mg/L of the four quinolones was analyzed using several mobile phases by varying the proportion of 1-propanol in the interval 2.5–12.5% *v*/*v*. The elution strength of the mobile phase increased at higher proportions of 1-propanol. In all the assays, the order of retention times was the same OXO < CIPRO < ENRO < SARA, and OXO was eluted far from the other ones. Low proportions of 1-propanol resulted in broad peaks and an excessive duration of the run. Otherwise, at higher proportions, OXO was eluted too close to the dead time and the resolution CIPRO/ENRO and ENRO/SARA diminished. The optimal value was set to 7.5%.

Finally, the detection conditions were set. A wavelength program was introduced by changing the excitation/emission wavelengths during the run in order to determine each quinolone at its maximal signal-to-noise ratio of emitted intensity. Therefore, the initial conditions were 260/366 nm (optimal values for OXO) and were maintained until 10.0 min (far from both the endpoint of OXO peak and the starting point of CIPRO peak. Then, the wavelengths were shifted to 280–455 nm, the optimal conditions of the other quinolones which were kept until the end of the chromatogram. Neither baseline drift nor baseline noise were affected by the wavelength change.

A system suitability test (SST) was performed by determining the experimental values of the main chromatographic responses/parameters under the optimized operating conditions. The results can be seen in Table 1. The values complied with the acceptance criteria [35]. Additionally, the excitation and emission spectra were registered at five points of the peak signal: maximum height, and at front and tail, 0.5 and 0.1 maximal height. For each quinolone, the spectra were alike thus proving the stability of the signal. Therefore, the chromatographic separation was successfully achieved.

The four antibiotics did not overlap and were reliably distinguished. Besides, OXO was eluted distantly enough to the dead time to prevent its overlapping with the front of the chromatogram. Moreover, the analysis time was not excessively long.

The antimicrobial drugs were resolved using a mobile phase essentially made of innocuous and biodegradable reagents. Only a low proportion of hazardous and volatile organic solvent (7.5%) was employed, less than usually required in hydroorganic HPLC (up to 100%). The SDS-micelles interacted with 1-propanol molecules and reduced its volatility, making the mobile phase more stable and less toxic. The four compounds were resolved using the isocratic mode, despite the different hydrophobicity and charges. This avoided the problems of baseline shift, spurious peaks from solvent impurities or gases, solvent demixing, and limited reproducibility of the chromatographic response (due to the difficulties in maintaining constant the separation conditions through several injections, as the proportion of solvent pumped to the system changes and the homogenization of the mixture may not be complete) inherent to gradient approaches. Besides, there was no need of re-equilibration time for the column thus shortening the effective analysis time per injection.

### 3.2. Sample Preparation

Sample processing and optimization of the experimental conditions were separately carried out for liquid and solid egg-derived foodstuffs. In both cases, a sample spiked at 0.5 mg/kg with the four studied antimicrobial drugs was used.

#### 3.2.1. Liquid Samples

The liquid egg-derived samples contained large amounts of non-water-soluble macromolecules, such as proteins and, in the case of those containing yolk, also fats, blended to other nutrients and the quinolones. The sample pretreatment was based on those detailed in References [30,31] but using the mobile phase as a dilution solvent instead of a pure micellar solution. When the sample was mixed with the hybrid micellar solution, the quinolones and some of the biological macromolecules were solubilized by interaction with the SDS micelles. The remaining macromolecules formed insoluble aggregates that were dispersed through the solution, but they were easily removed at the filtration step.

Several dilution ratios (1:1; 1:2; 1:5; 1:10; 1:25, and 1:50, *v*/*v*) were assayed. The values 1:1 and 1:2 were discarded as the filter was blocked early and the obtained volume was very low. In the other cases, more than 2 mL were collected. The matrix was eluted as a broad band in which the tallness decreased at higher dilution ratios at the front of the chromatogram, but the signal fell to the baseline before the elution of OXO. Therefore, 1:5 was chosen in order to obtain a reasonable sensitivity.

#### 3.2.2. Solid Samples

The sample pretreatment was an SBSLE, similar to those described in References [7,32], but using mobile phase as an extraction solvent for the same reasons as in Section 3.2.1. The quinolones and some of the macromolecules and micro-particles moved from the solid to the liquid phase during the stirring phase. The ultrasonication step was introduced to achieve this process and reduce the size of the particles which were then removed by filtration. Two factors, the stirring time and the sample-to-extractant solution (S/E, *w*/*v*) ratio, were optimized. The absolute recovery was determined by the peak area, corrected by the S/E ratio.

The effect of the stirring time was investigated over the range 5 min to 2 h. The absolute recovery rose from up to 30 min, slowly increased to 50 min, and remained nearly invariant to 2 h. Consequently, the stirring time was set to 60 min.

Several S/E ratios were tested: 1:1; 1:2; 1:5; 1:10; 1:25, and 1:50. For 1:1 and 1:2, the mixture was a viscous paste which could not be stirred, and then was directly rejected. The absolute recovery was nearly similar for the other values. In all cases, the matrix compounds were eluted at a broad band at the front of the chromatogram, but without overlapping with OXO. The optimal value was set to 1:5 in order to maximize the sensitivity.

#### 3.2.3. General Comments on the Procedure

In both cases, the sample pretreatment included the minimum number of easy-to-conduct steps, some of them semi-automated. The diluting/extracting solutions were directly injected in the column hence avoiding additional tedious and time- and resources-consuming extraction or purification steps and chemical reactions. Participation of the operator was minimal. A derivatization was not needed, as the quinolones exhibited natural fluorescence. Consequently, the probability of loss of the analyte and contamination of the sample during the preparation was strongly diminished. This contributed to ameliorating the reliability and consistency of the results.

The most used reagents were widely obtainable, stable, biodegradable, and innocuous. Only a minimal volume of toxic and volatile organic solvent was used. All the laboratory material and apparatuses required were general and accessible, as no specific devices, complex assembly or hyphenated extraction techniques were used.

### 3.3. Method Validation

The method was validated following the guidelines of the European Commission Decision 2002/657/EC [33], specifically developed to determine organic residues in foodstuff, and other documents regarding validation [35,38]. Because several and physico-chemically different kinds of samples were studied, the general parameters (instrumental calibration range and sensitivity, and ruggedness) were determined in standard solution, while the most specific ones (specificity, method calibration range and sensitivity, trueness, precision, decision limit, detection capability, and stability) were determined in each matrix.

#### 3.3.1. Instrumental Calibration Range and Linearity 

Standard solutions (*n* = 7) containing increasing concentrations of OXO, CIPRO, ENRO, and SARA (up to 0.2 mg/L) were analyzed in triplicate. The variances of the response (peak area) at the different levels were found significantly equivalent by an F-test, and thus the residuals of the calibration curve can be considered homoscedastic.

The average peak area was plotted versus the corresponding concentration. Both variables were related by a first-grade equation using the least squares regression method [38,39]. Table 2 shows the constants (slope and y-intercept and their respective standard deviations, SD), the goodness-of-fit parameters (determination coefficient, *R*^2^, and relative residual standard deviation, RRSD), outliers study (standardized residuals, ε_R_/s_y/x_, and Cook’s squared distance, CD^2^), and the sensitivity parameters (limit of detection, LOD, and limit of quantification, LOQ: as 3.3 and 10 times the deviation standard of the blank, taken as the standard deviation of the y-intercept, divided by the slope). No constant error was found, as the y-intercept was significantly equal to 0. Normality of the residuals was checked by the visualization of the plot residual versus concentration. According to the results, a proper linear relationship was demonstrated between the observed and independent variable in the range LOQ to 0.2 mg/L. The method limit of detection and quantifications (MLOD abd MLOQ, respectively) are the minimal amount detectable and quantifiable, respectively, in the sample. They are calculated from the LOD and LOQ considering the operations involved in the treatment). 

#### 3.3.2. Ruggedness

The effect of small changes in the operating conditions on the main chromatographic responses (retention time and peak area) was investigated for each quinolone using a Youden approach [33]. Minimal (x) and maximal (X) values were set at the same distance from the nominal value (the optimal one) to those that can be reached in the usual laboratory work (preparation of the mobile phase and random instrumental fluctuation). Studied factors and the x and X values were: (A) SDS concentration, 0.045–0.055 M; (B) 1-propanol, 7.3–7.7%; (C) TEA, 0.4–0.6%; (D) pH, 2.8–3.2; (E) flow rate, 0.95–1.05 mL/min; (F) injection volume, 18–22 μL; and (G) emission wavelength ± 5 nm. The assays were carried out using a standard solution of 0.1 mg/L of each antibiotic. The difference in response between the extreme values was judged significant if >5%.

Modification of the flow rate (>7.3%) and TEA (>6.8%) caused a change in the retention time while that of the injection volume on the peak area (>8.6%) for the four studied quinolones. The concentration of SDS influenced the retention time for OXO, ENRO, and SARA (>6.5%) and the peak area for ENRO (5.4%). The proportion of 1-propanol affected the peak area for ENRO (5.3%). In the other cases, the response parameters remained nearly unchanged. Therefore, the method can be considered robust enough to provide consistent values of chromatographic responses, although the main instrumental parameters underwent slight oscillations from their optimal value.

#### 3.3.3. Specificity

The ability of the method to discriminate OXO, ENRO, CIPRO, and SARA from endogenous compounds in the studied egg-derived samples was evaluated by analyzing blank samples and blank samples fortified with the four analytes at 0.5 mg/kg. The concentration of the quinolones in the injected solutions was 0.1 mg/L as the standard solution analyzed for the optimization and the SST (described in Section 3.1). In the fortified samples, the excitation and emission spectra were taken for the four analytes as described in Section 3.1. In all cases, a wide band (probably containing most of the macromolecules from the matrix) was eluted at the front of the chromatogram, from the dead time to nearly 3.0 min. No peaks from the endogenous compounds were observed close (±2.0 min) at the window time of the quinolones, and the baseline was quite stable.

**Figure 1:**
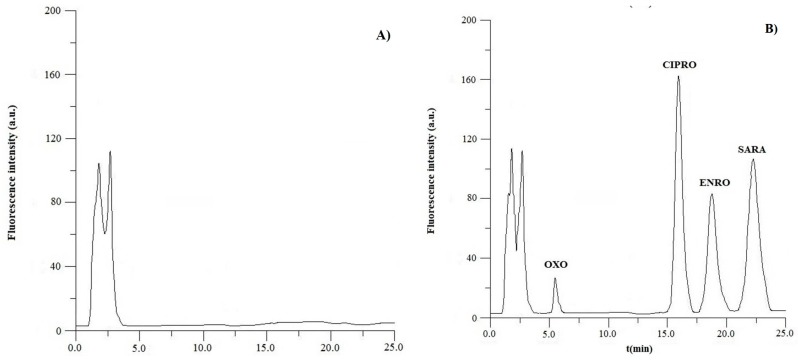
Chromatograms obtained by analysis of a sample of fresh egg yolk: (**A**) blank and (**B**) fortified with 0.5 mg/kg of each quinolone.

In the fortified samples, the retention time and the peak width at half-maximum height of the quinolones were similar (<3% and 96–103%, respectively) to that obtained from the analysis of the standard solution (Section 3.1). In order to evaluate peak purity, the chromatograms and the excitation and emission spectra, respectively, obtained from fortified samples and from the standard solution were overlaid and compared by visual observation. No significant differences were perceived among them thus assessing the absence of coeluting substances and matrix effects. The chromatogram obtained by the analysis of the fresh egg yolk (blank and spiked) is shown in Figure 1.

Using the here-described experimental conditions, each analyte gave the same response regardless of the original chemical environment, and no overlapping or coeluting compounds were noticed in the egg-derived foodstuff matrices. Therefore, this method is specific enough to reliably confirm the absence or presence of the analytes and to enable their quantification (as the entire peak area can be assigned to the corresponding quinolone) in an incurred egg-derived sample. This was mainly because of the strong association of the macromolecules (fats and proteins) with the micelles which prevent their interaction with the quinolones or the modified stationary phase and the use of natural fluorescence which limits the number of potential interfering substances.

#### 3.3.4. Method Calibration Range and Sensitivity

These parameters refer to the amount of quinolone that can be found in the unprocessed egg-derived sample. The method limit of detection (MLOD), the method limit of quantification (MLOQ), and the method upper limit of quantification (MULOQ) were calculated from the instrumental ones, considering the sample treatment. The values were the same for all the studied samples. The results can be seen in Table 2. We can see that even extremely low amounts of quinolone residues can be detected in the incurred samples.

As no permitted limit was stated by the EU Regulation 37/20108, a minimum required performance limit (MRPL) was fixed for each quinolone at their corresponding MLOQ. 

#### 3.3.5. Trueness and Precision

These parameters were separately determined for each quinolone and type of egg-derived foodstuff at three concentrations: 1×, 1.5×, and 2× MRPL. The trueness was determined by the consecutive analysis of six blank egg-derived samples spiked at the corresponding concentration. It was calculated as the difference between the average found concentration and the fortified concentration, divided by the fortified concentration, times 100. The repeatability was calculated from the same experiment as the relative standard deviation (RSD) of the six values of found concentrations. The same approach was made five days over a 3 month period (using freshly prepared samples in each case) to evaluate the consistency of the precision through time. The within-laboratory reproducibility was the RSD of the five average found concentrations. The results can be seen in Table 3.

The values (−14.2 to +9.8% for trueness and (<14.0% for precision) comply with the acceptance criteria stated by the guideline. Therefore, the method provides reliable quantitative data at concentrations close to the MRPL and inside the calibration curve. This performance was reached because of the simplicity of the sample preparation and the stability of the MLC responses. 

#### 3.3.6. Decision Limit and Detection Capability

The meaning of these parameters, stated by the EU Commission Decision 2002/657/EC, has been extensively described previously [33,38]. Both parameters were determined for each quinolone as indicated for substances for which no established limit has been stated, separately for each kind of egg-derived foodstuff.

The decision limit (CCα), with a α = 1%, was three times the standard deviation of the found concentrations obtained by the analysis of 20 blank samples. As the values were close to the MLOD, this value was taken as the CCα. Therefore, samples wherein the found concentration was over the MLOD were considered as non-compliant with the regulation, limiting to 1% the probability of rejecting a compliant sample.

The detection capability (CCβ) was calculated for β = 5% as the CCα plus 1.64 times the standard deviation of the detected concentrations by the analysis of 20 blank samples fortified at the decision limit. The results can be seen in Table 4. Samples containing antibiotic concentrations over these values have a low probability (<5%) to be accepted. The classification was barely affected by the uncertainty due to the closeness of the detection capability to the MLOD (<40%).

According to the results, this method is able to correctly evaluate the compliance with the regulation of egg-derived foodstuff samples at even low concentrations of quinolone residues.

#### 3.3.7. Stability

The stability of these antibiotics in working solutions kept in a fridge (+4 °C) in the darkness (at least two months) and bench-top in the micellar solution, obtained from a foodstuff sample pretreatment, at indoor conditions (a minimum of one day) has been proven in previously published studies [7,31,32]. Therefore, we examined the possible decay of the quinolones in the samples during storage at the usual conditions (in a freezer at −20 °C and in darkness).

For each kind of studied egg-derived foodstuff, a blank material was taken and divided into 20 parts which were fortified at 0.5 mg/kg of OXO, CIPRO, ENRO, and SARA. One aliquot was immediately analyzed and the other ones stored. These were analyzed each half week. The stability of the antimicrobial group in the stored samples was monitored by comparing the peak area corresponding to each quinolone with that obtained in the analysis of the fresh one as well as by the emergence of other peaks from decomposition products. No significant decay was noticed during the study period, and the incurred samples could be kept up to two months before analysis.

### 3.4. Comparison with Previously Published Methods

**The analytical performances of previously published procedures are summarized in Table 5.** We can see that the sensitivity of the present method was lower than that of the other methods. This is caused by the absence of a preconcentration step and the use of FLD instead of MS. Trueness and precision are clearly better than using MS, while they are similar to those based on FLD. The run time is longer, but this is compensated for with the short duration of the sample processing. The cost and the complexity of the entire procedure (considering the devices used in the sample pretreatment and the chromatographic instrumentation) are clearly inferior in the proposed method as well as the global use of organic solvent. This is clearly the strong point of the procedure.

### 3.5. Analysis of Incurred Samples

The analytical method was used for the screening of samples taken from fresh eggs (white and yolk) and industrially made egg products (boiled egg white and yolk; pasteurized egg white and yolk; powdered whole egg; powdered egg white and yolk; and omelet) (5 each one) purchased from local supermarkets to detect OXO, CIPRO, ENRO, and SARA residues. The analytes were not found in any sample thus confirming that they complied with the regulation on the absence of veterinary residues and could be ingested without risk by consumers.

The entire set of samples (20 liquid, 30 solid, and 7 for the calibration curve) was analyzed in one day by a single operator who could prepare, simultaneously, the solutions, perform the experimental protocol, control the instrumentation, and supervise the entire process. Certainly, the entire chromatographic sequence lasted <23 h. Liquid samples were treated in less than 1–2 min. Solid samples required a longer time (<90 min) but several samples can be simultaneously processed. Besides, the treatment of the samples can be performed while the previous ones are injected.

A large number of samples can be analyzed per day, resulting in a high sample throughput. In addition, the method can be executed with a relatively low disbursement (only common reagents, material, and instrumentation are used), taking into account the cost per studied sample. The solutions used have a limited toxicity (far less than usual in hydro-organic HPLC) and do not represent a risk for the environment and workplace safety. Consequently, the method can be used for routine analysis in a quality-control laboratory with a high workload.

## 4. Conclusions

The screening of samples from fresh eggs and the most common industrially made egg products to detect the antibiotics OXO, CIPRO, ENRO, and SARA is feasible using micellar liquid chromatography coupled with a fluorescence detector.

The main advantage is the sample pretreatment was easy-to-handle and required minimal participation from the operator, despite the physico-chemical complexity of the food matrices. The main chromatographic responses were checked by a system suitability test and were quite consistent. The analytical quality was evaluated by the guidelines of the EU Commission Decision 2002/657/EC (specificity, calibration range, linearity, trueness, precision, decision limit, detection capability, robustness, and stability). According to the results, the method was found effectively able to reliably identify and quantify the analytes in the studied matrices, reaching relatively low concentrations (0.025–0.150 mg/kg). The procedure exhibited interesting practical features, such as eco-friendliness, safeness, inexpensiveness, wide availability, and a high sample throughput, which make it useful for screening purposes. These outstanding practical and analytical performances were attained because of the particular properties of the micellar solutions used for diluting/extracting solutions and mobile phases. Therefore, the procedure can be implemented in laboratories approved for official residue control to evaluate the compliance of egg-derived samples with the EU regulation 37/2010 regarding the occurrence of the studied antimicrobials.

## Figures and Tables

**Table 1:** System suitability testing. Main chromatographic parameters obtained under the optimal conditions (*n* = 6). (t_R_: retention time; RSD: relative standard deviation; OXO, oxolinic acid; CIPRO, ciprofloxacin; ENRO, enrofloxacin; SARA, sarafloxacin).

Parameter	OXO	CIPRO	ENRO	SARA	Acceptance Criteria
t_R_ (min) (RSD, %)	5.48 ± 0.05 (0.9)	16.08 ± 0.14 (0.9)	18.79 ± 0.17 (0.9)	5.48 ± 0.05 (0.9)	16.08 ± 0.14 (0.9)
RSD of peak area, %	0.6	0.9	0.5	0.7	<1.0
RSD of peak width at half-maximum height, %	0.5	0.8	0.7	0.9	<1.0
Retention factor	4.48	15.08	17.79	21.3	>2.0
Efficiency	2172	2842	2584	2158	>2000
Asymmetry	1.4	1.2	1.2	0.9	0.8–1.6
Resolution with the next eluted quinolone	High	1.9	2.4	-	>1.5

**Table 2:** Calibration and sensitivity parameters (SD: standard deviation; RRSD: relative residual standard deviation; *r*^2^: determination coefficient; ε_R_: residual; s_y/x_: residual standard deviation; CD: Cook’s squared distance; LOD: limit of detection; LOQ: limit of quantification; MLOD: method limit of detection; MLOQ: method limit of quantification).

Parameter	OXO	CIPRO	ENRO	SARA
Slope ± SD	1353 ± 5	16,970 ± 30	8240 ± 20	10,760 ± 80
y-Intercept ± SD ^a^	−7 ± 4	18 ± 8	6 ± 4	−12 ± 16
*r* ^2 b^	0.9991	0.9994	0.9997	0.9992
RRSD (%) ^c^	1.3	0.8	0.6	0.9
Maximum ε_R_/s_y/x_^d^	1.2	0.8	0.5	1.1
Maximum CD^2 e^	0.8	0.4	0.3	0.8
LOD (μg/L)	10	2	2	5
LOQ (μg/L)	30	5	5	15
MLOD (μg/kg)	50	10	10	25
MLOQ (μg/kg)	150	25	25	75
Method calibration range (mg/kg)	0.15–1.0	0.025–1.0	0.025–1.0	0.075–1.0

Acceptance criteria: ^a^ t(5; 0.05; 2 tails) × SD; ^b^ >0.990; ^c^ <1.5; ^d^ <3; ^e^ <1.

**Table 3:** Trueness (%), repeatability ^1^, and within-laboratory reproducibility ^2^ (RSD, %) values for the quantification of the studied quinolones in fresh eggs and several egg-derived foodstuff (acceptance criteria: trueness, −20 to +10%; precision, <15.1%). For each matrix, the first, second, and third lines refer to the fortified amount MRPL, 1.5× MRPL, and 2× MRPL).

Sample	OXO	ENRO	CIPRO	SARA
Fresh egg yolk	+7.5/6.2/7.0	−6.9/7.1/6.8	−10.7/9.3/9.2	−9.3/8.9/10.6
	+6.5/6.1/7.2	−4.0/5.2/4.7	−7.8/8.2/7.9	−8.5/10.5/9.5
	+4.2/4.8/5.3	−2.9/4.8/6.0	−6.5/7.3/6.5	−4.2/6.2/6.5
Fresh egg white	+7.3/6.9/6.7	−7.0/6.5/8.0	−9.9/9.6/9.0	−8.9/8.5/8.7
	+5.2/6.2/7.6	−4.9/6.0/7.2	−7.5/6.8/7.2	−7.8/7.0/6.8
	+5.0/5.9/7.1	−2.6/3.9/4.4	−5.9/6.8/6.4	−5.5/6.0/6.9
Boiled egg yolk	+9.5/8.0/9.8	−9.8/8.5/8.9	−14.2/13.3/12.5	−13.8/14.0/12.9
	+7.9/7.1/8.0	−7.2/6.8/7.0	−9.5/8.3/7.7	−10.0/12.2/11.8
	+6.8/4.9/6.4	−5.6/7.2/6.8	−6.9/7.1/5.8	−7.5/8.5/7.2
Boiled egg white	+9.2/7.4/8.7	−9.4/9.0/10.2	−13.8/13.4/14.0	13.2/11.4/13.9
	+7.0/6.8/8.1	−6.9/5.9/6.8	−10.5/9.2/8.9	−9.7/10.2/11.0
	+4.9/5.0/5.8	−4.8/4.3/5.2	−7.5/8.0/8.6	−7.0/6.5/7.3
Pasteurized egg yolk	+6.9/7.2/7.5	−6.5/7.2/7.5	−9.7/8.5/7.9	−8.9/9.3/9.8
	+5.0/4.8/5.9	−4.3/6.8/7.0	−7.9/7.5/8.0	−7.5/6.7/7.0
	+3.9/4.0/4.9	−3.0/4.9/5.0	−6.2/6.0/6.5	−5.2/6.5/5.8
Pasteurized egg white	+6.8/6.8/7.2	−7.0/6.9/7.2	−9.2/7.8/8.2	−8.6/9.0/9.4
	+5.8/6.1/6.0	−5.0/6.2/6.3	−8.2/7.4/7.8	−7.2/7.3/6.9
	+4.0/3.8/3.6	−3.4/4.2/4.7	−6.1/6.4/6.9	−4.9/5.2/5.7
Powdered egg yolk	+9.5/8.2/8.4	−10.9/9.9/10.6	−13.5/12.8/11.9	−12.9/10.0/10.9
	+7.5/7.0/6.5	−8.0/8.2/7.9	−11.0/9.9/10.5	−9.4/8.5/8.1
	+5.2/5.0/5.8	−5.9/5.8/6.0	−7.3/7.5/7.2	−7.3/7.0/7.4
Powdered egg white	+9.8/8.5/8.9	−8.2/7.9/8.5	−13.6/12.1/12.8	−12.9/11.5/12.0
	+7.1/6.8/7.2	−6.7/7.1/8.0	−8.8/9.2/8.7	−8.3/8.0/7.5
	+5.4/4.8/5.3	−4.2/6.3/6.7	−5.8/6.8/7.0	−5.0/6.3/5.2
Powdered whole egg	+9.2/8.0/7.8	−9.9/10.2/10.8	−12.8/11.7/10.3	−12.6/10.6/9.8
	+7.8/6.9/7.0	−8.3/8.5/8.0	−10.5/9.8/9.1	−9.2/8.7/7.9
	+5.6/5.8/5.3	−6.1/6.4/6.0	−6.9/7.4/8.0	−7.2/6.5/7.0
Omelet	+8.9/7.6/9.0	−11.0/10.8/11.6	−14.0/13.2/12.9	−13.6/10.3/12.5
	+6.8/8.5/7.5	−7.8/8.0/7.5	−10.9/10.3/9.5	−9.7/8.2/9.0
	+5.0/6.2/6.8	−5.8/6.5/5.8	−7.0/8.5/7.8	−7.8/6.9/7.5

^1^*n* = 6; ^2^ *n* = 5.

**Table 4:** Detection capacity for each quinolone in the studied meats (concentrations in μg/kg).

Sample	OXO	ENRO	CIPRO	SARA
Fresh egg yolk	56	12	12	30
Fresh egg white	57	11	12	29
Boiled egg yolk	61	12	13	33
Boiled egg white	60	13	13	33
Pasteurized egg yolk	58	13	12	28
Pasteurized egg white	57	12	12	29
Powdered egg yolk	63	13	13	34
Powdered egg white	62	12	13	33
Powdered whole egg	64	13	12	35
Omelet	63	13	14	34

**Table 5:** Practical and analytical performances of previously published methods.

Reference	Sample Treatment (Volume of Organic Solvent)	Organic Solvent in Mobile Phase (%)	Instrument	LOQ (μg/kg)	Trueness (%)	Precision (RSD, %)	Run Time (min)
This paper	Dilution or STLE (very low)	1-propanol and TEA (8)	HPLC-FLD	50	85–110	<14	25
10	STLE+LLE+SPE (high)	Acetonitrile (10)	HPLC-FLD	8	87	5	13
12	STLE (low)	Methanol (70)	SPME-HPLC-FLD	8	89–106	7	20
18	QuEChERS™ (low)	Acetonitrile (up to 95%)	UPLC-MS-MS	0.2	78–105	8	11
19	STLE+protein precipitation+SPE (medium)	Methanol (up to 100%)	UPLC-MS-MS	5	59–98	17	12
20	STLE (low)	Acetonitrile (up to 90%)	HPLC-MS	1.5	78–110	15	20
23	STLE+LLE (medium)	Acetonitrile (up to 25%)	HPLC-FLD	30	89	15	25
25	STLE+SPE (medium)	Acetonitrile (up to 35%)	HPLC-MS	25	88–121	22	26
27	STLE+LLE (medium)	Acetonitrile (up to 40%)	HPLC-FLD	25	86–107	14	20

SPE: solid phase extraction; SMPE: solid phase micro-extraction; STLE: solid-to-liquid extraction; LLE: liquid/liquid extraction: QuEChERS: quick, easy, cheap, effective, rugged, and safe™ solid-phase extraction procedure; UPLC: ultra-performance liquid chromatography.
